# Supplementary material for: Unveiling the Bioactive Potential of the Invasive Jellyfish Phyllorhiza punctata Through Integrative Transcriptomic and Proteomic Analyses
Source: Biomolecules. 2025 Aug 4;15(8):1121. doi: 10.3390/biom15081121 (PMC12383608; doi:10.3390/biom15081121)
Supplement: Supplementary file 1 [file biomolecules-15-01121-s001.zip › Suplementary Material/FileS3/PEPFOLD4/XP_065063046_14078/clusters.html]

 PEP-FOLD clusters


Download archive of all models  here   

|  |  |  |
| --- | --- | --- |
| # | file | sOPEP |
| # cluster 1 - Size: 3 |  |  |
|  | XP\_065063046\_14078-model1 | -93.3708 |
|  | XP\_065063046\_14078-model1.1 | -83.5282 |
|  | XP\_065063046\_14078-model1.2 | -77.9409 |
| # cluster 2 - Size: 1 |  |  |
|  | XP\_065063046\_14078-model2 | -92.7524 |
| # cluster 3 - Size: 3 |  |  |
|  | XP\_065063046\_14078-model3 | -87.358 |
|  | XP\_065063046\_14078-model3.1 | -73.5499 |
|  | XP\_065063046\_14078-model3.2 | -72.3131 |
| # cluster 4 - Size: 2 |  |  |
|  | XP\_065063046\_14078-model4 | -87.0371 |
|  | XP\_065063046\_14078-model4.1 | -72.0959 |
| # cluster 5 - Size: 1 |  |  |
|  | XP\_065063046\_14078-model5 | -86.7734 |
| # cluster 6 - Size: 5 |  |  |
|  | XP\_065063046\_14078-model6 | -84.3347 |
|  | XP\_065063046\_14078-model6.1 | -66.0605 |
|  | XP\_065063046\_14078-model6.2 | -63.3197 |
|  | XP\_065063046\_14078-model6.3 | -61.6017 |
|  | XP\_065063046\_14078-model6.4 | -60.7864 |
| # cluster 7 - Size: 1 |  |  |
|  | XP\_065063046\_14078-model7 | -82.2181 |
| # cluster 8 - Size: 1 |  |  |
|  | XP\_065063046\_14078-model8 | -80.9556 |
| # cluster 9 - Size: 2 |  |  |
|  | XP\_065063046\_14078-model9 | -80.4472 |
|  | XP\_065063046\_14078-model9.1 | -58.5385 |
| # cluster 10 - Size: 1 |  |  |
|  | XP\_065063046\_14078-model10 | -80.3672 |
| # cluster 11 - Size: 1 |  |  |
|  | XP\_065063046\_14078-model11 | -78.2348 |
| # cluster 12 - Size: 2 |  |  |
|  | XP\_065063046\_14078-model12 | -76.5766 |
|  | XP\_065063046\_14078-model12.1 | -65.4256 |
| # cluster 13 - Size: 1 |  |  |
|  | XP\_065063046\_14078-model13 | -76.4168 |
| # cluster 14 - Size: 1 |  |  |
|  | XP\_065063046\_14078-model14 | -76.1815 |
| # cluster 15 - Size: 1 |  |  |
|  | XP\_065063046\_14078-model15 | -75.1543 |
| # cluster 16 - Size: 2 |  |  |
|  | XP\_065063046\_14078-model16 | -74.906 |
|  | XP\_065063046\_14078-model16.1 | -60.2005 |
| # cluster 17 - Size: 1 |  |  |
|  | XP\_065063046\_14078-model17 | -74.4863 |
| # cluster 18 - Size: 1 |  |  |
|  | XP\_065063046\_14078-model18 | -73.9093 |
| # cluster 19 - Size: 1 |  |  |
|  | XP\_065063046\_14078-model19 | -73.5412 |
| # cluster 20 - Size: 2 |  |  |
|  | XP\_065063046\_14078-model20 | -72.2623 |
|  | XP\_065063046\_14078-model20.1 | -67.8484 |
| # cluster 21 - Size: 1 |  |  |
|  | XP\_065063046\_14078-model21 | -71.7962 |
| # cluster 22 - Size: 1 |  |  |
|  | XP\_065063046\_14078-model22 | -70.7665 |
| # cluster 23 - Size: 1 |  |  |
|  | XP\_065063046\_14078-model23 | -70.3357 |
| # cluster 24 - Size: 2 |  |  |
|  | XP\_065063046\_14078-model24 | -69.1616 |
|  | XP\_065063046\_14078-model24.1 | -61.0208 |
| # cluster 25 - Size: 1 |  |  |
|  | XP\_065063046\_14078-model25 | -68.5275 |
| # cluster 26 - Size: 1 |  |  |
|  | XP\_065063046\_14078-model26 | -68.1056 |
| # cluster 27 - Size: 1 |  |  |
|  | XP\_065063046\_14078-model27 | -67.1174 |
| # cluster 28 - Size: 2 |  |  |
|  | XP\_065063046\_14078-model28 | -66.7914 |
|  | XP\_065063046\_14078-model28.1 | -60.5386 |
| # cluster 29 - Size: 1 |  |  |
|  | XP\_065063046\_14078-model29 | -66.5282 |
| # cluster 30 - Size: 1 |  |  |
|  | XP\_065063046\_14078-model30 | -66.3355 |
| # cluster 31 - Size: 1 |  |  |
|  | XP\_065063046\_14078-model31 | -65.8961 |
| # cluster 32 - Size: 2 |  |  |
|  | XP\_065063046\_14078-model32 | -65.8546 |
|  | XP\_065063046\_14078-model32.1 | -57.5454 |
| # cluster 33 - Size: 1 |  |  |
|  | XP\_065063046\_14078-model33 | -65.047 |
| # cluster 34 - Size: 1 |  |  |
|  | XP\_065063046\_14078-model34 | -65.0459 |
| # cluster 35 - Size: 3 |  |  |
|  | XP\_065063046\_14078-model35 | -64.793 |
|  | XP\_065063046\_14078-model35.1 | -62.7954 |
|  | XP\_065063046\_14078-model35.2 | -50.9806 |
| # cluster 36 - Size: 3 |  |  |
|  | XP\_065063046\_14078-model36 | -64.6754 |
|  | XP\_065063046\_14078-model36.1 | -59.5645 |
|  | XP\_065063046\_14078-model36.2 | -58.8835 |
| # cluster 37 - Size: 2 |  |  |
|  | XP\_065063046\_14078-model37 | -64.6087 |
|  | XP\_065063046\_14078-model37.1 | -64.1034 |
| # cluster 38 - Size: 1 |  |  |
|  | XP\_065063046\_14078-model38 | -64.4819 |
| # cluster 39 - Size: 1 |  |  |
|  | XP\_065063046\_14078-model39 | -64.2711 |
| # cluster 40 - Size: 1 |  |  |
|  | XP\_065063046\_14078-model40 | -64.2228 |
| # cluster 41 - Size: 2 |  |  |
|  | XP\_065063046\_14078-model41 | -63.8227 |
|  | XP\_065063046\_14078-model41.1 | -62.9483 |
| # cluster 42 - Size: 1 |  |  |
|  | XP\_065063046\_14078-model42 | -63.7113 |
| # cluster 43 - Size: 1 |  |  |
|  | XP\_065063046\_14078-model43 | -63.313 |
| # cluster 44 - Size: 1 |  |  |
|  | XP\_065063046\_14078-model44 | -62.3226 |
| # cluster 45 - Size: 1 |  |  |
|  | XP\_065063046\_14078-model45 | -62.0338 |
| # cluster 46 - Size: 1 |  |  |
|  | XP\_065063046\_14078-model46 | -61.2612 |
| # cluster 47 - Size: 1 |  |  |
|  | XP\_065063046\_14078-model47 | -61.1462 |
| # cluster 48 - Size: 1 |  |  |
|  | XP\_065063046\_14078-model48 | -60.166 |
| # cluster 49 - Size: 3 |  |  |
|  | XP\_065063046\_14078-model49 | -59.8214 |
|  | XP\_065063046\_14078-model49.1 | -57.9563 |
|  | XP\_065063046\_14078-model49.2 | -56.9841 |
| # cluster 50 - Size: 1 |  |  |
|  | XP\_065063046\_14078-model50 | -59.7499 |
| # cluster 51 - Size: 1 |  |  |
|  | XP\_065063046\_14078-model51 | -59.6374 |
| # cluster 52 - Size: 1 |  |  |
|  | XP\_065063046\_14078-model52 | -59.6296 |
| # cluster 53 - Size: 1 |  |  |
|  | XP\_065063046\_14078-model53 | -59.4508 |
| # cluster 54 - Size: 1 |  |  |
|  | XP\_065063046\_14078-model54 | -59.1898 |
| # cluster 55 - Size: 1 |  |  |
|  | XP\_065063046\_14078-model55 | -58.8706 |
| # cluster 56 - Size: 1 |  |  |
|  | XP\_065063046\_14078-model56 | -58.7913 |
| # cluster 57 - Size: 1 |  |  |
|  | XP\_065063046\_14078-model57 | -58.6901 |
| # cluster 58 - Size: 1 |  |  |
|  | XP\_065063046\_14078-model58 | -57.8624 |
| # cluster 59 - Size: 1 |  |  |
|  | XP\_065063046\_14078-model59 | -57.2301 |
| # cluster 60 - Size: 1 |  |  |
|  | XP\_065063046\_14078-model60 | -57.1864 |
| # cluster 61 - Size: 1 |  |  |
|  | XP\_065063046\_14078-model61 | -56.7132 |
| # cluster 62 - Size: 1 |  |  |
|  | XP\_065063046\_14078-model62 | -56.3431 |
| # cluster 63 - Size: 2 |  |  |
|  | XP\_065063046\_14078-model63 | -56.2023 |
|  | XP\_065063046\_14078-model63.1 | -54.4834 |
| # cluster 64 - Size: 1 |  |  |
|  | XP\_065063046\_14078-model64 | -56.0517 |
| # cluster 65 - Size: 1 |  |  |
|  | XP\_065063046\_14078-model65 | -56.0126 |
| # cluster 66 - Size: 1 |  |  |
|  | XP\_065063046\_14078-model66 | -54.3277 |
| # cluster 67 - Size: 1 |  |  |
|  | XP\_065063046\_14078-model67 | -54.3205 |
| # cluster 68 - Size: 2 |  |  |
|  | XP\_065063046\_14078-model68 | -54.31 |
|  | XP\_065063046\_14078-model68.1 | -53.4725 |
| # cluster 69 - Size: 1 |  |  |
|  | XP\_065063046\_14078-model69 | -53.5834 |
| # cluster 70 - Size: 1 |  |  |
|  | XP\_065063046\_14078-model70 | -53.1016 |
| # cluster 71 - Size: 1 |  |  |
|  | XP\_065063046\_14078-model71 | -52.11 |
| # cluster 72 - Size: 1 |  |  |
|  | XP\_065063046\_14078-model72 | -51.7422 |
| # cluster 73 - Size: 1 |  |  |
|  | XP\_065063046\_14078-model73 | -50.6702 |
| # cluster 74 - Size: 1 |  |  |
|  | XP\_065063046\_14078-model74 | -47.4574 |
|  |  |  |
